# Evolution and Critics on “Capnography as an Aid in Localizing the Phrenic Nerve in Brachial Plexus Surgery. Technical Note” by Combined Ventilator Waveforms Analysis

**DOI:** 10.1055/s-0037-1608623

**Published:** 2017-10-30

**Authors:** George Georgoulis, Eirini Papagrigoriou, Marc Sindou

**Affiliations:** 1 Department of Neurosurgery, University of Lyon 1, Hôpital Neurologique “Pierre Wertheimer,” Lyon, France; 2 Department of Neurosurgery, University of Athens Medical School, Athens, Greece; 3 Intensive Care Unit, “Agios Panteleimon” General Hospital of Nikaia, Piraeus, Greece; 4 Department of Neurosurgery, University of Lyon 1, Lyon, France

Dear Editor,

We have recently published a work on the intraoperative identification of fourth cervical (C4) root and phrenic nerve during “difficult” surgery, by changing the ventilator waveforms triggered by electrical stimulation of these anatomic structures. 1 2 Reviewing the literature on the domain, we came across the correspondence by Bhakta (October 2008) regarding the article “Capnography as an aid in localizing the phrenic nerve in brachial plexus surgery. Technical note.” 3 4 Having studied the method in a series of 12 patients for C4 root (microsurgical cervical DREZotomy for neuropathic pain after brachial plexus avulsion) and 2 patients for phrenic nerve (transversomegaly of seventh cervical vertebra and brachial plexus tumor), we would like to add our own findings and conclusions in the discussion.

In the original article (May 2008), Bhagat et al 4 had presented their work in which the changes on capnography elicited by electrical stimulation of the phrenic nerve had been successfully used for the intraoperative identification of the nerve in a series of three patients. In October 2008, Bhakta questioned the correlation between the changes on capnography and the stimulation, suggesting that various anesthesiologic parameters could have resulted in similar changes.

Our own method consists of the combined analysis of capnography and at least one of pressure–time and flow–time curves. In our series, general intravenous anesthesia was used, without neuromuscular blocking agents. As opposed to the patients in the series of Bhagat et al, where a laryngeal mask was used, 4 our patients were intubated and ventilated in fully controlled ventilation modes with tidal volumes of 6 mL/kg and frequencies between 11 and 15. No difference of performance was observed between volume control and pressure control modes. No hyperventilation or air leak around the cuff was suspected at any point. No poststimulation hemodynamic variability was observed. The electrical stimulation was always performed at around 1 mA.

Under these fully controlled conditions, capnography showed a sensitivity of 100% in the detection of the stimulation of either C4 root or phrenic nerve.

**Fig. 1:**
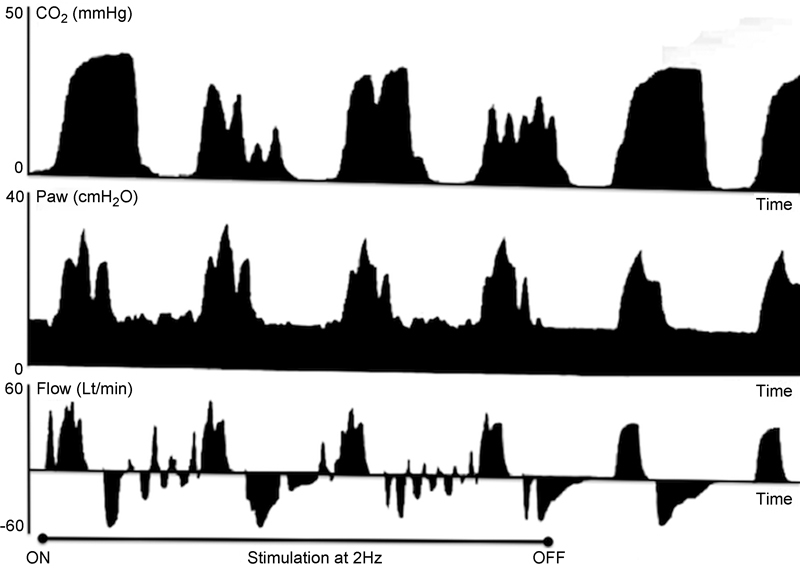
Intraoperative stimulation of phrenic nerve at 1 mA and 2 Hz in a patient operated for upper brachial plexus trunk tumor (schwannoma). Combined study of the three curves: (1) The onset and end of changes on pressure and flow curves are concomitant with the onset (ON) and end (OFF) of stimulation on pressure and flow curves. Capnography lags behind by a few seconds. (2) Sharp nonspecific descends on capnography. Successive dentations on pressure–time and miniature respiratory cycles on flow–time curves, compatible with repetitive partial contractions of the diaphragm. (3) The calculation of frequency is easier during expiration and may necessitate freezing the screen for a few seconds. In our example, ventilator frequency = 11 breaths/minute; respiratory cycle = 60 seconds/12 = 5 seconds; inspiratory:expiratory time ratio = 1:2, thus inspiratory time = 5 seconds × 1/3 = 1.7 seconds and expiratory time = 5 seconds × 2/3 = 3.3 seconds. On flow–time curve, six responses on average are seen during expiration; thus, their frequency is 6/3.3 seconds ≈ 2/seconds, a close approximation of the frequency of our electrical stimulation of the phrenic nerve at 2 Hz (two cycles per second).

The disadvantages of the capnography curve alone are that it can only be interpreted during expiration, as values during inspiration are zero, and there is normal delay between the occurrence of ventilatory events and their appearance on the curve. The combined analysis of the three curves offered valuable additional information, thus increasing the specificity of the findings. The study of pressure and flow curves, which are real-time curves covering the entire respiratory cycle, allowed us to confirm the on–off effect, that is, the appearance and disappearance of the changes concomitantly with the onset and end of stimulation. The patterns observed on capnography were of greater amplitude but rather nonspecific, whereas those observed on pressure and/or flow curve were generally of smaller amplitude but more specific, often resembling miniature respiratory cycles. Yet the key feature was that the patterns on pressure and/or flow curves were repetitive, although not always uniform, corresponding to the frequency of the stimulation. This was not so obvious on capnography, where changes were mostly cumulative. In fact, in some patients it was possible to calculate the frequency of these miniature patterns, which were found to coincide with the frequency used (in our practice 2 Hz). We thus believe that the morphology, repetitiveness, and frequency of these patterns can rule out false positives or artifacts of any kind ( Fig. 1 ).

This method, whether it employs capnography alone or, preferably, capnography combined with pressure/time or flow/time or both, requires no modification in the anesthesia or surgery protocol or on any additional equipment. It is perfectly innocuous and feasible in all circumstances. It is our firm belief that it is a simple, reliable, and useful tool in the cervical spine and brachial plexus surgery.

Best regards,
